# Design of Metal-Organic Framework Templated Materials Using High-Throughput Computational Screening

**DOI:** 10.3390/molecules25214875

**Published:** 2020-10-22

**Authors:** Momin Ahmad, Yi Luo, Christof Wöll, Manuel Tsotsalas, Alexander Schug

**Affiliations:** 1 Steinbuch Centre for Computing, Karlsruhe Institut für Technologie, Herrmann-von-Helmholtz Platz 1, 76344 Eggenstein-Leopoldshafen, Germany; momin.ahmad@kit.edu; 2 Institute of Functional Interfaces, Karlsruhe Institut für Technologie, Herrmann-von-Helmholtz Platz 1, 76344 Eggenstein-Leopoldshafen, Germany; yi.luo@partner.kit.edu (Y.L.); christof.woell@kit.edu (C.W.); 3 Institute for Advanced Simulation, Jülich Supercomputing Center, Wilhelm-Johnen-Straße, 52428 Jülich, Germany; 4 Faculty of Biology, University of Essen-Duisburg, Universitätsstr. 5, 45141 Essen, Germany

**Keywords:** metal-organic frameworks, cross-linker, cross-linking, high-throughput screening, molecular dynamics, simulation

## Abstract

The ability to crosslink Metal-Organic Frameworks (MOFs) has recently been discovered as a flexible approach towards synthesizing MOF-templated “ideal network polymers”. Crosslinking MOFs with rigid cross-linkers would allow the synthesis of crystalline Covalent-Organic Frameworks (COFs) of so far unprecedented flexibility in network topologies, far exceeding the conventional direct COF synthesis approach. However, to date only flexible cross-linkers were used in the MOF crosslinking approach, since a rigid cross-linker would require an ideal fit between the MOF structure and the cross-linker, which is experimentally extremely challenging, making in silico design mandatory. Here, we present an effective geometric method to find an ideal MOF cross-linker pair by employing a high-throughput screening approach. The algorithm considers distances, angles, and arbitrary rotations to optimally match the cross-linker inside the MOF structures. In a second, independent step, using Molecular Dynamics (MD) simulations we quantitatively confirmed all matches provided by the screening. Our approach thus provides a robust and powerful method to identify ideal MOF/Cross-linker combinations, which helped to identify several MOF-to-COF candidate structures by starting from suitable libraries. The algorithms presented here can be extended to other advanced network structures, such as mechanically interlocked materials or molecular weaving and knots.

## 1. Introduction

**Figure 1:**
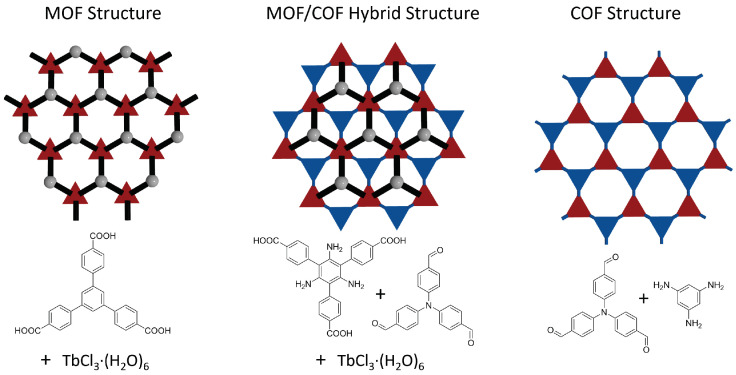
Schematic representation of the conversion of a MOF to a COF. The first step is to synthesize the MOF. The hybrid MOF/COF structure is the cross-linked MOF. The COF is achieved by removing the metals out of the structure.

Network topology, i.e., the way (molecular) building blocks are connected along three dimensions, critically influences key properties of natural and synthetic materials [1,2,3]. The advent of crystalline “framework” materials allowed to realize the long dreamed-of possibility to control materials on the molecular level [4]. Metal-Organic Frameworks (MOFs) [5] and Covalent-Organic Frameworks (COFs) [6] are prime examples of such “materials on demand”. MOFs and COFs share common features such as porosity and crystallinity. COFs feature attractive properties, such as extended π-conjugation and being composed of purely light weight elements [7,8]. Yet, despite considerable progress in developing COFs [9], the structural variability of MOFs is considerable larger, as MOFs are able to leverage the vast chemical parameter space of their organic and inorganic components. Previously, MOFs have been hybridized with covalent polymer networks [10,11,12,13] or were used to template polymer synthesis by taking advantage of the large variability of MOFs. So far, no attempts were reported to combine the topological variability of MOFs with the fascinating features of COFs within one material. A scheme depicting such a MOF-COF hybrid structure and potential components is provided in Figure 1. Realizing the combination of two frameworks within such a MOF-COF hybrid material would, however, require careful selection of the involved components as both frameworks are inherently rigid and do not permit adjustments via partially flexible components. Currently, systematic experimental efforts to identify such combinations are not feasible as they would rely on lucky guesses via trial-and-error approached. Thus, an effective way for high-throughput in silico design appears as indispensable and without alternative.

The growing capabilities of high-performance computing (HPC) combined with continuously expanding databases of existing MOFs suggest that high-throughput in silico screening could offer a low-cost strategy to identify promising MOF structures [3,14,15,16,17,18]. In related fields such as structural biology, computational techniques like Molecular Dynamics take advantage of HPC and have lead to significant new insights [19,20,21]. For MOF, high-throughput computational screening focuses on popular research fields such as hydrogen uptake [16,17], methane adsorption [15], or growth mechanism [3]. Recently also geometrical considerations were investigated, such as macroscale heteroepitaxial alignment [18]. By considering more and more complex secondary building units, the possibilities to synthesize a MOF, and thus, the number of theoretical possible MOFs rose drastically [4]. At the same time, considering larger databases of possible MOFs, hinges on improving the efficiency of high-throughput computational methods to evaluate structures without a loss of reliability. Other approaches of cross-linking are absed on retrofitting MOFs by interconnecting open-metal sites [22,23]. Important physical properties, such as the mechanical stability, can be improved by functionalizing the organic linker of a MOF [24] and benefit from analysis of a large MOF database to identify suitable partners. For example, introducing a secondary network besides the primary framework that further stabilizes the framework can be achieved by cross-linking as mentioned above. However, our approach differs in that instead of introducing an additional linker binding to open-metal sites, the cross-linkers are docked directly onto the organic linkers to form covalent bonds. Hence, we are not dependent on any open-metals sites, and this method allows us to freely choose binding sites on any given organic linker (of course under consideration of the chemistry). Furthermore, the final material contained two frameworks in one system (MOF and COF) and could ultimately lead to synthesizing MOF-templated COF structures, thereby representing an innovative use of high-throughput screening methods.

In this work we focus on cross-linking the organic linkers of a MOF [25]. There are two imaginable ways to find compatible MOF-cross-linker pairs, either first choosing a MOF and then screening a cross-linker library or first choosing a cross-linker and then screening a library of different MOF types. The first approach would require a database of cross-linkers. However, in a given MOF only very few cross-linking paths are possible. Since these are not known a priori, this approach requires the construction of a very large database of cross-linkers of variable length to increase the probability of finding any matches. Such a large database of cross-linkers, to the best knowledge of the authors, does not exist and would first have to be generated. Hence, if currently the goal is to find the optimal cross-linker for a desired MOF, one has to fall back to manually designing the cross-linkers. In the present work we focus on the second approach described above. In this case we can make use of the fact that there exist multiple large MOF databases comprising millions of potential crosslinking paths suitable for any given cross-linker length. Therefore, in this paper we developed such a high-throughput screening tool to identify MOF “perfect fits” for a given cross-linker, by employing the CoReMOF-database [26]. The CoReMOF-database is a collection of experimentally synthesized MOF crystal structures reported in literature. The presented screening method relies on geometric factors, namely distances, angles and rotations. Especially possible rotations of linkers are interesting and have been the focus of many investigations [27,28,29,30,31]. After suitable MOF candidates have been identified their combination with the given cross-linker is then further analyzed by Molecular Dynamics (MD) simulations to refine the structure.

## 2. Results and Discussion

### 2.1. Overview Computational Approach and Screening Concept

A cross-linker connects two or more linkers of a MOF. Choosing the reaction type and the functionalization of the linker allows one to place the cross-linker in specific desired positions. Whether a chosen cross-linker fits into a certain MOF structure can be guided by (a) experience (Which often, even for experimentalists with large experience with MOFs, boils down to educated guessing), (b) experimentally testing many MOF/cross-linker combinations or (c) calculating the actual relevant sizes and manually selecting suitable leads. The options (a) and (b) become quickly expensive and are very time-consuming. Automatizing the third option (c), i.e., to calculate all distances to achieve the maximum amount of matches for a given cross-linker, appears attractive to improve the probability of finding good candidates. Another benefit of calculating geometries is that additional factors such as chemical bond angles or different orientations or even conformations of the linker can be included in a second filtering process to achieve “perfect fits”.

To screen a library of MOFs, its content needs to contain all crucial data of the structure, such as coordinates and cell parameters, it needs to be updated frequently to reflect our growing knowledge of MOFs, and it needs to be properly annotated for analysis. The CoReMOF-database has been developed with high-throughput analysis in mind and was already successfully exploited by other groups [18]. The MOF structures are stored as CIF-files (short for Crystallographic Information Framework) and can be read by a python module called *pymatgen* [32]. In particular, pymatgen also allows to search for bonds, which is crucial to identify potential binding sites, making the CoReMOF-database an ideal base for this work.

**Figure 2:**
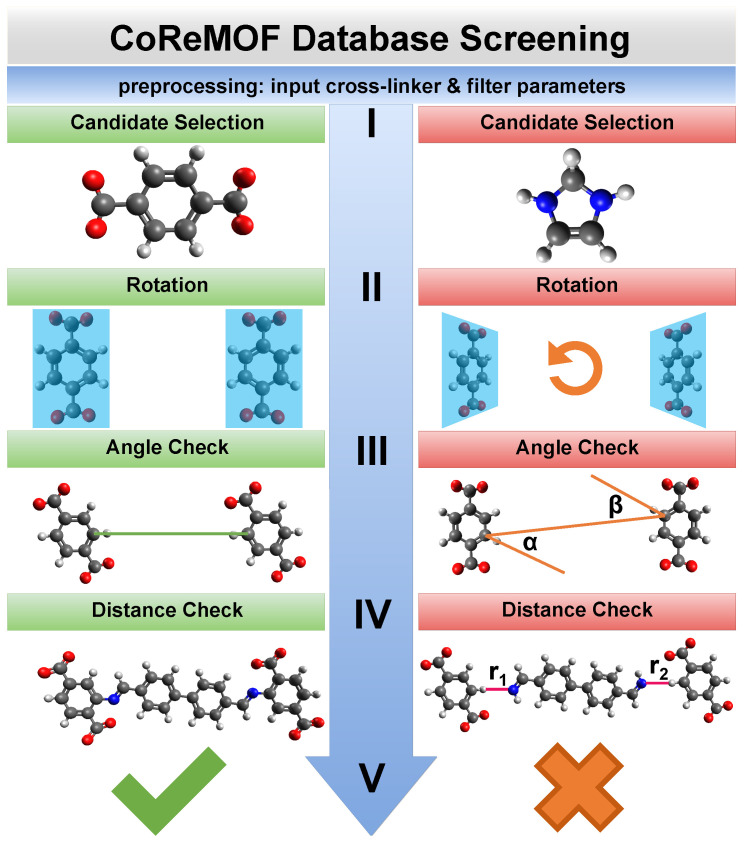
Flowchart of the screening process: (left) Ideal case of a MOF (right) structures that do not fulfill the selection criteria. In the preprocessing the parameters for the cross-linker and filters are set as input values. The first step (I) looks for all candidates. In our case these are hydrogen atoms from an aromatic ring. The second step (II) checks if a rotation is necessary. If so, the candidates are rotated towards each other. The next step (III) calculates the angles α and β that define the line of sight from one candidate to another (or to the geometric center). If these angles are above the angle limit, the structure is dismissed. The last filter (IV) checks if the cross-linker length fits between two (or more) candidates. Here, this is not the case on the right hand side where $r_{1}$ and $r_{2}$ represent too large bond lengths. The MOF is saved if all filters are passed (left) or dismissed otherwise (right) (V).

The screening algorithm is divided into several subsequent tasks of increasing complexity as shown in Figure 2. In this work only MOFs are included where a cross-linker binds to the organic ligand consisting of one or many aromatic rings. The organic linker bdc is such an example. The cross-linker in our case binds to an aromatic carbon of an organic linker with one or more carbon rings. This means the binding atom of the cross-linker replaces the hydrogen atom that is connected to said aromatic carbon atom. We call these hydrogen atoms candidates. At first, selection criteria such as desired cross-linker length, limits and step size are defined. The cross-linker length is determined as the distance of the two binding points or as the distance of a binding point to the geometric center for more than two binding points). Additionally, some cross-linker have several energy minima resulting from different possible conformations. Accordingly different lengths need to be considered. After that, suitable candidates are chosen from a given MOF that possess a defined structure (I), in this case organic linkers with carbon rings. The candidates are the atoms that are replaced by the binding sites of the cross-linkers, i.e., the connection points of a MOF for cross-linkers. One iteration of the screening compares the same number of candidates as the number of binding sites of the cross-linkers with each other. This includes the rotation of the organic linkers to each other, so that their distances are minimized (II), and the comparison of the ’line of sight’ between candidates (III). The line of sight is defined as the line that begins at the candidate’s neighboring atom and passes through the candidate himself. Since an atom can have multiple neighboring atoms, there can be several such lines. However, it will turn out that the candidates are hydrogen atoms and therefore these special cases need not be considered. If these lines (largely) overlap, it can be assumed that the cross-linker, inserted later, is not subject to any additional stress that could be caused by an unfavorable binding angle between cross-linker and MOF. The next step finally calculates the distance(s) between the candidates (IV) and compares them against the calculated cross-linker length(s) within a tolerance. The tolerance is expressed as an upper and lower limit related to the cross-linker length and can be freely defined. In the last step a positive hit is saved, a negative result is dismissed and the screening executes the next iteration (V). A negative result is achieved if (a) no candidates are found (see below), (b) the lines of sight deviate too much or (c) the cross-linker does not fit into the MOF. A hit occurs accordingly, if all filters are passed through without violating any selection criteria. More details including other pre-filters can be found in Materials and Methods.

Before predicting novel (potential) COF structures, we checked our code on an already synthesised cross-linked structure. This initial screening served as a test for our methodology. Finding the previously known structure demonstrates good performance of our approach.

### 2.2. Proof of Concept

**Figure 3:**
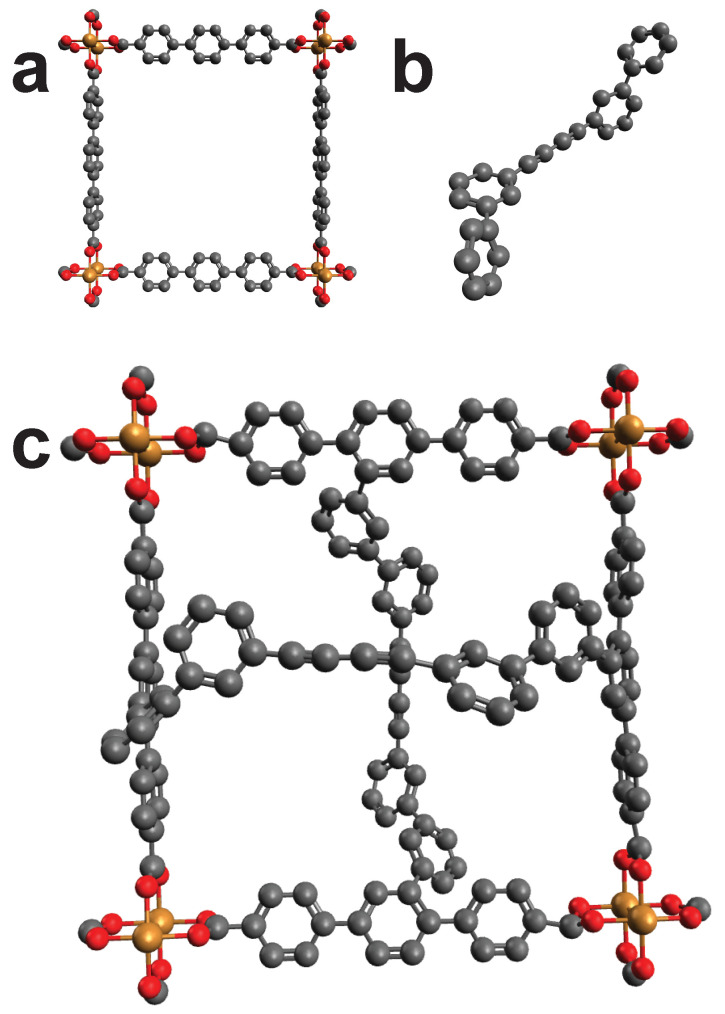
(**a**): First a frame for the MOF was created using AuToGraFS. (**b**): The linker was created again by the principle of handling the reaction partners as already reacted. (**c**): In this case the cross-linker is made up by two separate cross-linkers (that are not connected with each other) with in total four binding points. The cross-linker connects to the frame in a crossed shape and reflects the molecular weaving. Color code: Black (C), Red (O), Gold (Cu). Hydrogens omitted for visibility.

Wang et al. turned a surface mounted MOF (SURMOF) into an interwoven textile by “molecular weaving ” [33]. Basically a MOF was cross-linked to stabilize the structure. We pretend to not know this functionalization but only the two components that are meant to bind with each other. Using the length of the cross-linker and one frame of the MOF, we can benchmark our algorithm by screening the MOF. The algorithm should signal a HIT when screen the MOF frame by using the parameters of the cross-linker. Following steps were executed: first the unmodified MOF was created with AuToGraFS [34]. Then the cross-linker was separately created with Avogadro. For the screening, the cross-linker was seen as a single structure, thus, making it a cross-linker with four binding points. Figure 3 shows the created structures. Going through the database resulted in a HIT for the initial MOF frame, thus, confirming the ability of our methodology to identify good fits.

### 2.3. Screening of Cross-Linkers

**Figure 4:**
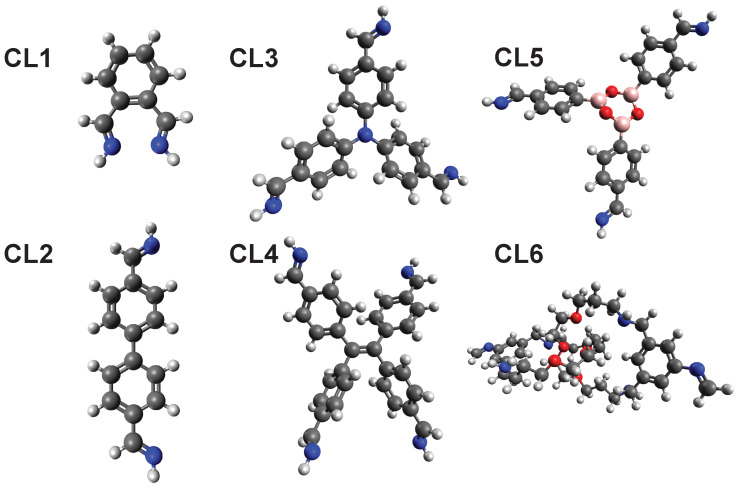
List of cross-linkers used for the screening. Color code: Hydrogen (White), Carbo (Black), Nitrogen (Blue), Oxygen (Red), Boron (Pink).

We screened six cross-linkers (CL1–CL6, cf. Figure 4) to find novel structures and prime candidates for future COFs. Cross-linker CL1 and CL2 are simple cross-linker with two binding points. CL3 is able to bind to three and CL4 binds to four organic linkers. CL5 and CL6 represent more exotic cross-linker showing the large scope of the presented screening approach. CL5 describes a ship-in-a-bottle approach, where the cross-linker can be generated within the MOF via connection of three components. CL6 represents an example of a mechanically interlocked molecule utilized as a cross-linker, potentially forming MOF-templated mechanically interlocked materials. The chemical structure for CL6 can be found in Figure 9. For the screening the CoReMOF-database of 11 April 2019 was used.

**Figure 5:**
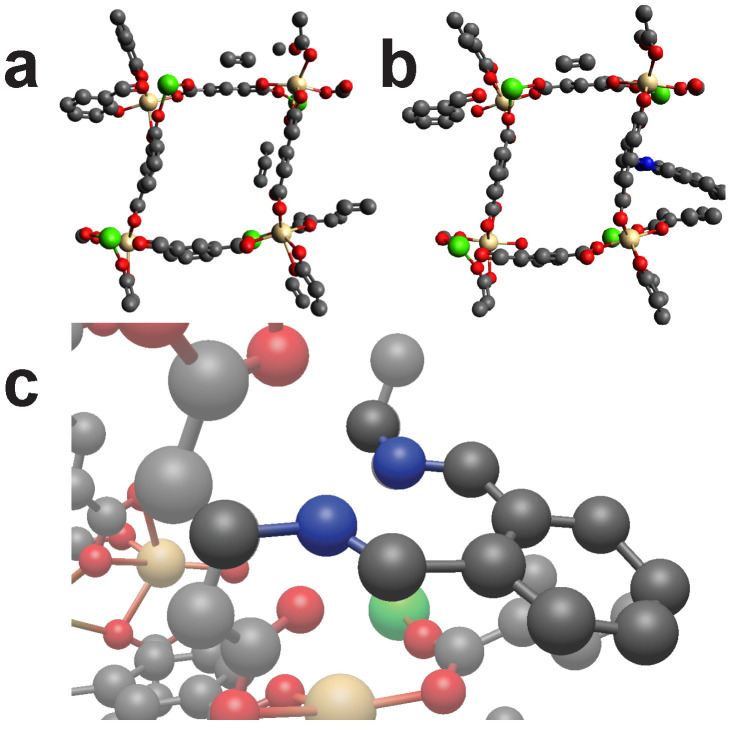
The MOF “EKOPEA” can be cross-linked by using cross-linker CL1. (**a**): Unmodified view of EKOPEA. (**b**): EKOPEA with cross-linker attached. (**c**): Zoomed view of cross-linking. Color code: Black (C), Blue (N), Red (O), Green(Ca), Gold (Cd). Hydrogens omitted for visibility.

In Figure 5 the MOF “EKOPEA” is cross-linked by CL1 that connects two neighboring linkers as identified by the screening process. The symmetry of the MOF allows the functionalization of all adjacent linkers and thus complete cross-linking. Higher order hierarchies are feasible.

**Figure 6:**
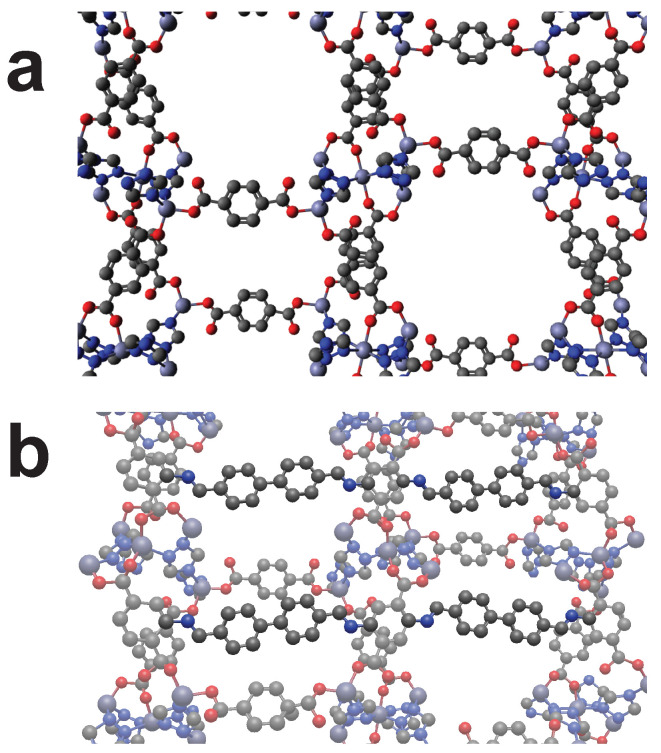
The MOF “IBUBIT” can be cross-linked by using cross-linker CL2. (**a**): Unmodified view of IBUBIT. (**b**): Cross-linked IBUBIT. Color code: Black (C), Blue (N), Red (O), Purple (Zn). Hydrogens omitted for visibility.

Another example for a good fit is the MOF “IBUBIT” that generated a HIT by using cross-linker CL2. Figure 6 shows an unmodified and modified version as suggested in our screening. Here again two adjacent linkers of the MOF are cross-linked, so that an ongoing functionalization throughout the MOF crystal may be possible.

**Figure 7:**
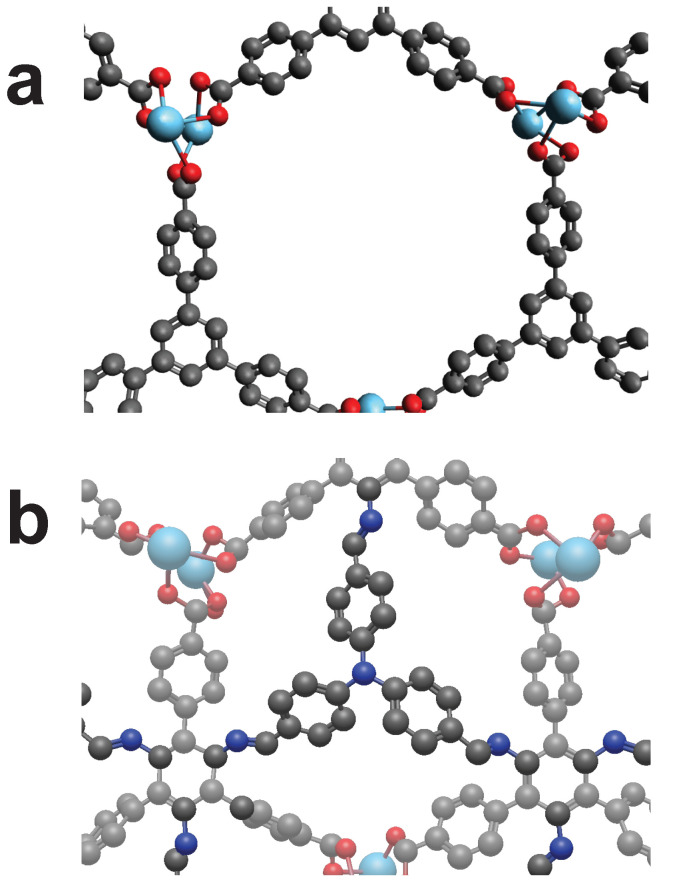
The MOF “RUYVEO” can be cross-linked by using the cross-linker CL3. (**a**): Unmodified view of RUYVEO. (**b**): Cross-linked RUYVEO. Color code: as above plus Lanthanung (Turquoise). Hydrogens omitted for visibility.

A very interesting hit is provided by the MOF “RUYVEO” that allows to be cross-linked by CL3 (Figure 7). It nicely fits into the ring like structure and connects to the same type of bond in all three cases. The linker possesses a ring structure, but no defined axis of rotation, as the carbon ring is connected thrice.

**Figure 8:**
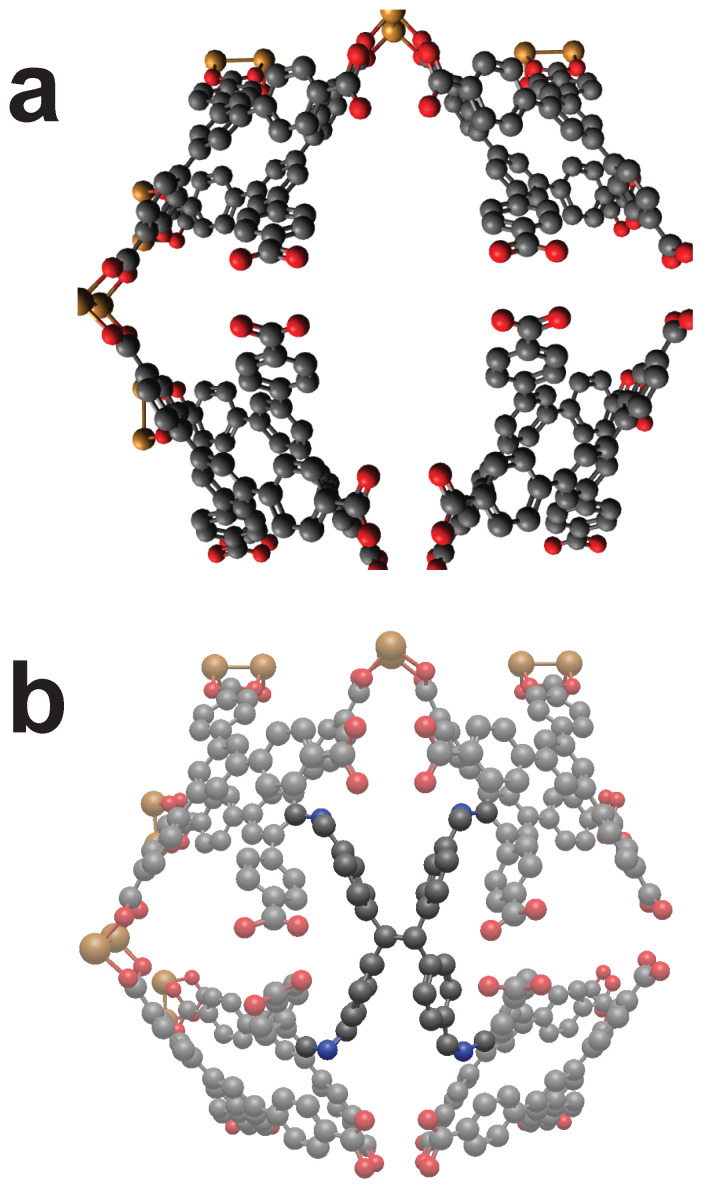
The MOF “QOWRAV12” can be cross-linked using cross-linker with four binding points. (**a**): Unmodified view of QOWRAV12. (**b**): Cross-linked QOWRAV12. Color code: as above plus Copper (Gold). Hydrogens omitted for visibility.

In Figure 8 another case where the linker of the MOF “QOWRAV12” is not able to rotate but still allows the functionalization by cross-linker CL4. Here, the cross-linker connects two halves, which are connected by copper atoms at another place.

### 2.4. Exotic Cross-Linkers Examples

**Figure 9:**
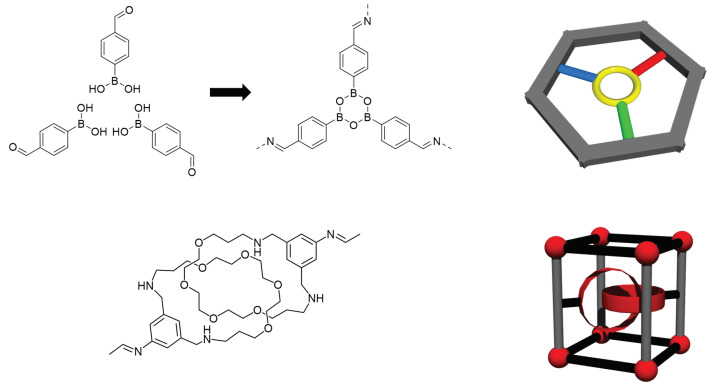
Catenane and “ship in a bottle” examples.

Cross-linker CL5 and CL6 are more a look into the future of this screening than being cross-linkers that may possibly be part of a COF. The examples show a possible “ship in a bottle” approach where the cross-linker forms in situ, as well as a mechanically interlocked structure, where in this example catenated molecules could be employed as the cross-linker. Figure 9 describes the concept of both approaches.

**Figure 10:**
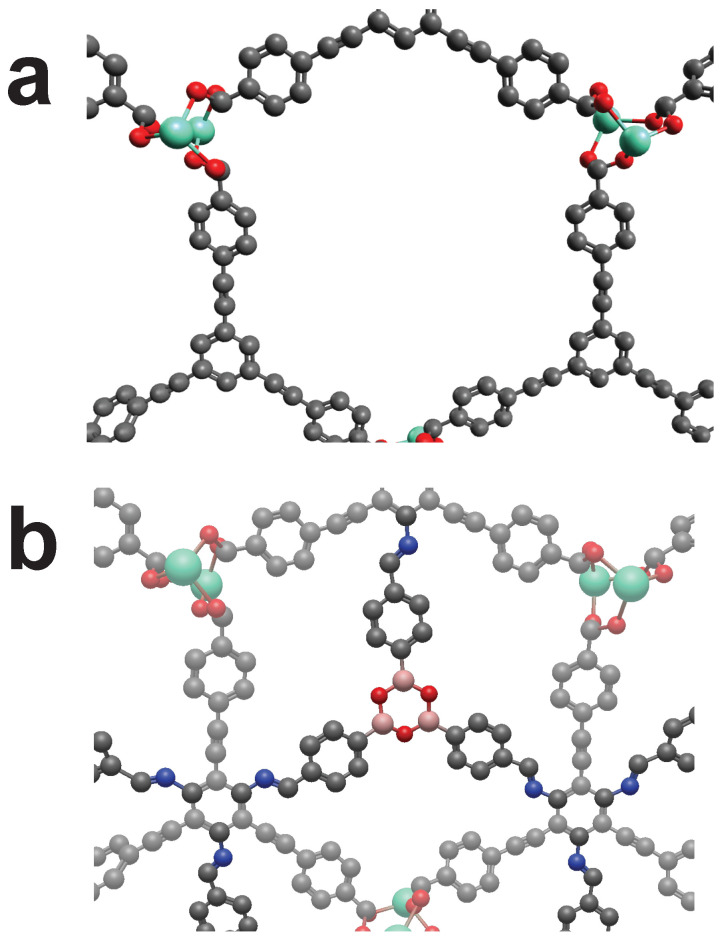
The MOF “RUYVIS” can be cross-linked by using cross-linker CL5. (**a**): Unmodified view of RUYVIS. (**b**): Cross-linked RUYVIS. Color code: Black (C), Blue (N), Red (O), Pink (B), Turquoise (Eu). Hydrogens omitted for visibility.

Both cross-linkers show that this screening method can be used to find different MOF structures for different purposes. In case of CL5 the screening finds a similar structure (see Figure 10) to the one found for cross-linker CL3. Here once again the advantage of screening becomes clear, as a distinction is made between similar MOFs and cross-linkers to find the best suiting partners.

**Figure 11:**
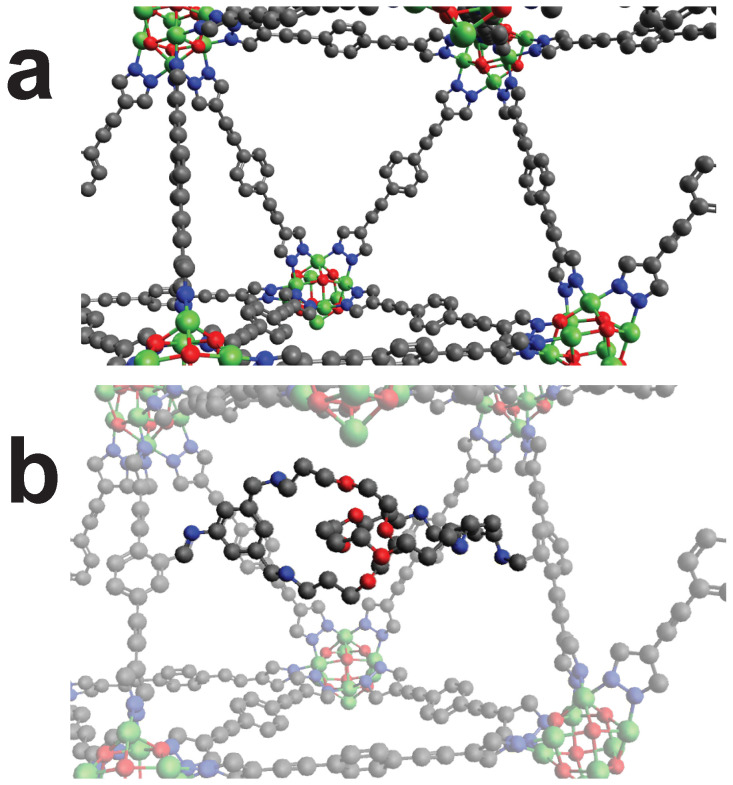
The MOF “BIBXOB” can be cross-linked by mechanically interlocking of two catenas. (**a**): Unmodified view of BIBXOB. (**b**): Cross-linked BIBXOB. Color code: Black (C), Blue (N), Red (O), Green (Ni). Hydrogens omitted for visibility.

The MOF “BIBXOB” shown in Figure 11 possesses a large unit cell and is able to host cross-linker CL6 into its cell. Two diagonally positioned linkers allow the cross-linking of this MOF. If every cell has such a cross-linker, a complete functionalization can be achieved.

### 2.5. Study of Two Bond Cross-Linkers

**Figure 12:**
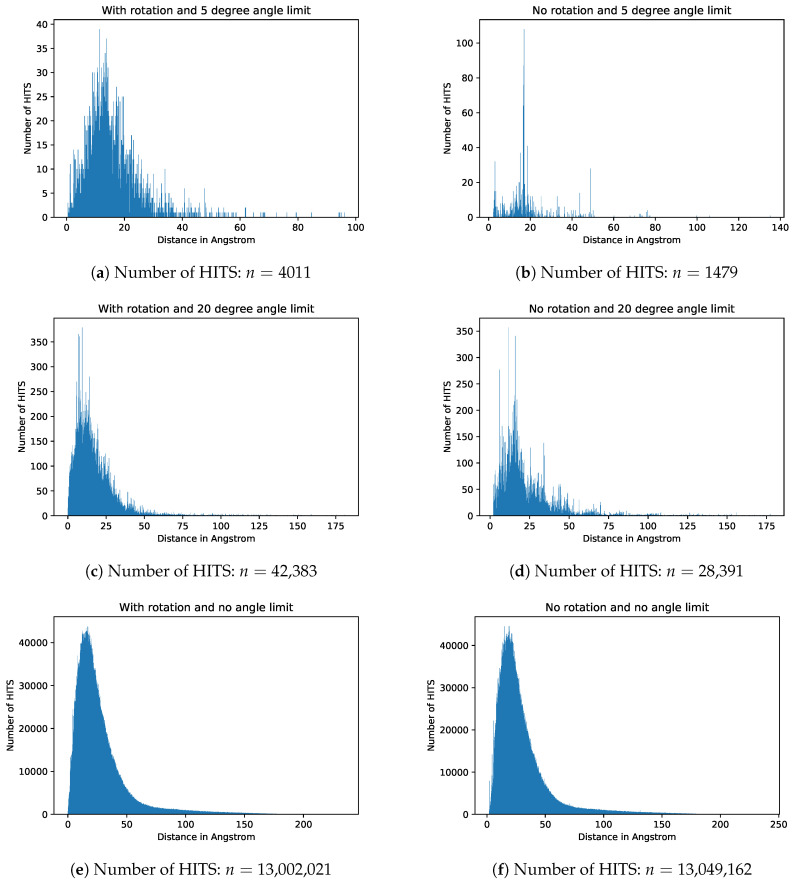
Screening of all occurring distances. Number of bins are ten times the maximum distance each. The individual figures (**a**–**f**) display different choices of screening parameters as labeled in the respective figure.

To obtain an overview of all occurring distances between two candidates, a screening without a defined length was performed with the angle limits being 5 degrees, 20 degrees and with no angle limit at all. Furthermore, a screening of the database was performed with the same angle limits but with the rotation of the linker disabled. The result is shown in Figure 12 in form of histograms.

As one can see, the number of possible connections increases with increasing distance until a peak around 17Å is reached. After that the number of possible connections decreases sharply and shows an asymptotic behavior towards larger lengths. Another interesting finding is that after rotation the number of possible contacts increases. One assumption could be, that if a MOF with a certain symmetry generates a HIT after rotation, then not only one but many more hits (depending on the symmetry) are generated. In the case of non-rotated linkers, many arbitrary connections are found where the candidates are placed across the whole MOF. With increasing angle the hits logically increase, because the angle filter allows more results. If all angles are allowed, the influence of rotation disappears accordingly.

### 2.6. Improvement Suggestions

The simplifications made in the screening algorithm can lead to ”false friends”. These are hits technically fulfilling all selection criteria but being in violation of chemical principles. For examples, if in Figure 15 the two unnamed carbon atoms from the aromatic ring would have been replaced by a single atom of any element, the filters would still allow this structure, as the algorithm completely disregards those two carbon atoms. Another example would be the bonds. If a ring structure possesses more than two binding sites towards the metal node (e.g., HKUST-1), the algorithm would still find candidates and a rotation axis when there should be none. The results in Figure 7, Figure 8 and Figure 10 highlight this special case. However, in these cases a rotation was not necessary and the results are still valuable. Nevertheless such cases should be treated separately. Another problem is that this screening algorithm does not check whether the path between the candidates is free of other atoms. One possible solution to this particular problem would be to include the tool Zeo++ during the screening pipeline, that checks for void space in the structure and is widely used in the MOF community [35]. In general, we currently recommend to check all hits manually for their chemical viability as any automatic procedure can fail.

## 3. Materials and Methods

The screening algorithm is composed by subsequent tasks of increasing complexity as shown in Figure 2. A hit occurs after passing all filters.

### 3.1. Candidate Selection

**Figure 13:**
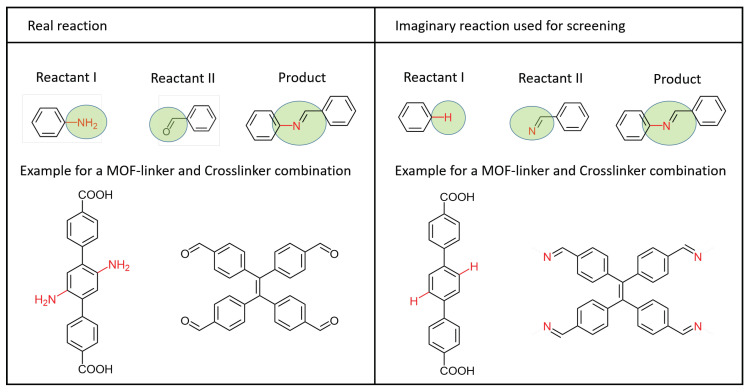
The laboratory conditions would be, as shown on the left, that the MOF is functionalized with an amine group. This amine group can then react with the oxygen of the cross-linker and form the MOF/cross-linker bond. However, the structures in the MOF database are stored in their unmodified form. To simplify our virtual screening, the cross-linker is assumed to carry the product of the reaction, and the MOF remains in its unmodified state, as shown on the right side. The atom in whose place the amine group was bound is now the atom that is replaced by the binding atom of the cross-linker.

For the synthesis of cross-linked MOFs the organic ligands of the MOF have to be functionalized to control the chemical reaction, i.e., cross-linkers have to be reactive to those functionalizations. As the CoReMOF-database contains mostly non-functionalized structures, scanning the original educts is problematic. One option would be to modify the entries in the database prior to screening and consider the chemistry of MOF binding during screening. We chose a less complex way to address this challenge by considering both reaction partners as “product carrying”: Instead of screening the database for the original cross-linker, the modified cross-linker contains all the relevant structure information of the reaction product, allowing us to screen within unmodified MOF structures where any hydrogen atom is a potential reaction partner. An example is shown in Figure 13. This simplification does not only address having to simulate the chemical reaction, but also is a more general model of a connection between linker and cross-linker and allows us to screen through the database quickly without having to consider the chemistry behind it.

**Figure 14:**
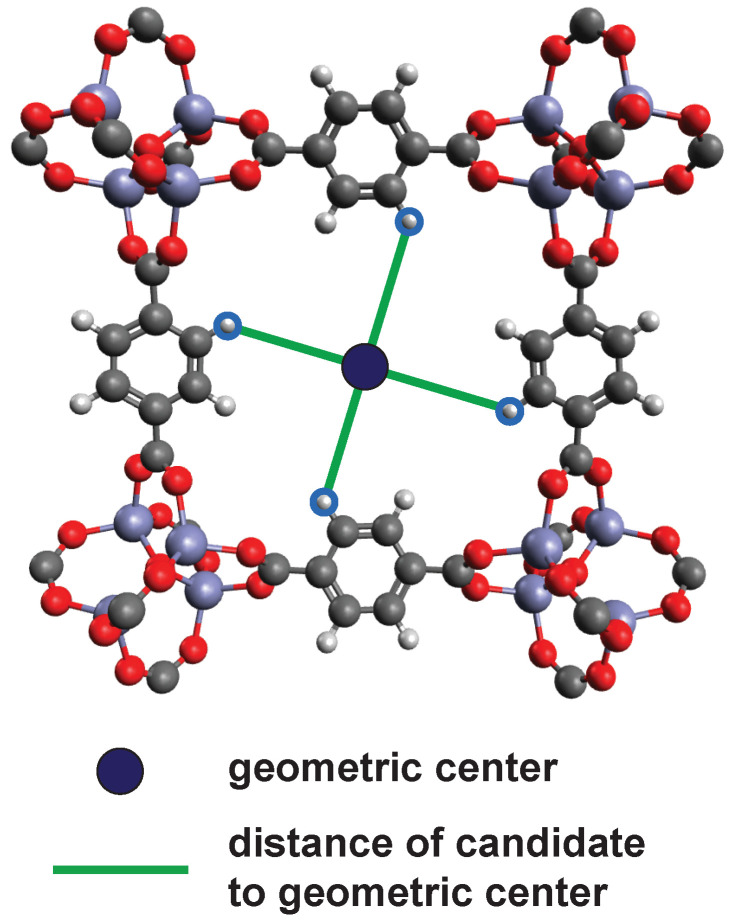
2D frame of the MOF MOF-5. If there are more than two binding sites, one does not calculated the distances between any two candidates. Instead, the distance of each candidate to the geometric center is calculated. since the same applies to a cross-linker with more than two bonds, the individual distances can be compared.

As mentioned in the screening concept, we only consider MOFs where a cross-linker binds to the organic ligand consisting of one or many aromatic rings and call the binding hydrogen atom on said aromatic rings candidates. The source code is modular and can be tailored. e.g., the part where candidates are selected can easily be changed to accommodate different linkers. If one wants to know if a certain cross-linker fits into a MOF, the distance between two of these candidates (in case of a linear cross-linker) has to be determined. This distance can then be compared against the length of the cross-linker. The method must be slightly modified for cross-linkers with more than three binding sites. In this case the distances from each candidate to another are no longer useful. First the geometric mass-weighted center of the candidates is calculated. Subsequently, the distances between the geometric center and the candidates can be calculated. The same is done for the cross-linker. Since as many candidates are compared as the cross-linker has binding sites, each distance of the cross-linker can be compared with a distance of the MOF. Figure 14 shows the geometric center of four candidates and the resulting distances. If, for example, a cross-linker with four binding points is placed in such a way that both geometric centers lie on top of each other and the individual distances from each other hardly differ, this would be a hit.

**Figure 15:**
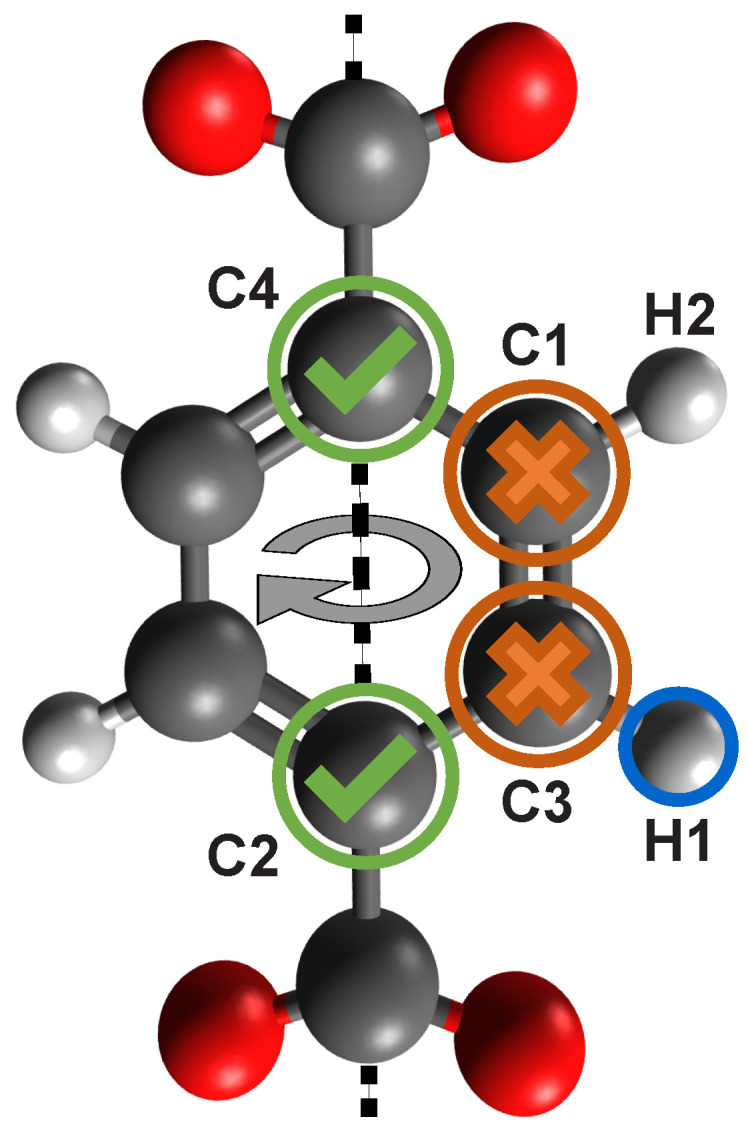
To identify a candidate all hydrogen atoms are first collected. Every hydrogen not connected to a carbon atom is disregarded. The screening algorithm then checks if the carbon atom is part of an aromatic ring structure and if a rotation axis exists by checking its neighbors in each direction. If a carbon atom is found that is connected to another three carbon atoms in each direction, the algorithm marks the hydrogen as a positive candidate.

Before the screening starts, all candidates of a structure must be identified. The following steps are visualized in Figure 15. The selection criteria for the candidates is as follows: first, search for all hydrogen atoms. Second, check if the hydrogen (H1) is connected to an aromatic carbon (C3) atom. For this step the algorithm looks for atoms around the hydrogen below a defined distance threshold of 1.55Å. Third, scan the binding partners of the carbon (C3) atom. If it is part of a ring structure, then it has to be connected to two other carbon atoms (C1 and C2) and to the first hydrogen (H1). In case of a ring structure one of the carbon atoms (C1 or C2) has to be connected again to two other carbons (C3 and C4) atoms and again one hydrogen (H2) atom, and the second carbon atom (C1 or C2) has to be connected to three other carbon atoms. In this example C2 is connected to three other carbon atoms, therefore, C3 has to be connected to another carbon atom (C4) that again has three carbon atoms as binding partners. If those two carbon atoms (C2 and C4) are found, a rotation axis exists and the hydrogen is saved as a candidate together with its neighboring atom (C1) and the carbon atoms (C2 and C4). The last two carbon atoms are essential to define the rotation axis.

### 3.2. Screening

Before the screening, obviously poor candidate pairs are dismissed (e.g., distances that are considerable too large ±20Å) without any more detailed analysis. Furthermore, to forbid cross-linking of the same linker twice, candidates must not share the same rotation axis, as then they would be part of the same linker. In case of three or more bonds, one can look for an initial symmetry by comparing each distance of hydrogen candidate to the center of mass. For example, if all distances from the geometric center to the binding point of the cross-linker are the same length, all combinations of MOF candidates that do not possess this property can be sorted out beforehand.

The rotation of the linker minimizes the distance between the candidates (or candidates to center of mass) to prevent unwanted twisting of the cross-linker, which could occur, for example, if the candidates are offset with respect to each other. After the rotation, the angle between carbon atom and hydrogen is checked. The angle is calculated by defining two lines. The first line is that connecting both carbon atoms (or carbon atom to geometric center) that neighbor the candidate, and the second line goes from said carbon atom to the candidate. The smaller the angle between those two lines, the more the hydrogen candidates are in-line. This minimizes the possibility of an unfavorable angle between MOF and cross-linker. Lastly, the distance between candidates candidates (candidate to geometric center) is calculated and compared to the distances of the cross linker. If it is in a specific range (flexible cross-linker results in a larger range), the screening reports a positive hit and writes the output into a log file. The range can be chosen at the beginning of the screening.

### 3.3. Structure Optimization

To confirm the positive hits identified in the screening process, a quick geometry optimization is executed after placing the cross-linker into the MOF. The cross-linker is positioned inside the MOF and via Molecular Dynamics (MD), the energy of the the modified structure is minimized. If the MOF structure is strongly deformed during the geometry-optimization the corresponding case is labeled as bad fit. Such deformations occur when binding points have to be maintained by the MD software and for this reason unnatural bond lengths, angles and torsion angles are formed (note that classical MD neither creates nor destroys pre-defined bonds). A perfect fit changes the overall structure only by a minimal amount. Tools that were used for the structure optimization were Avogaro [36] (version 1.2.0) and in some cases GULP [37,38,39,40]. The force-fields employed for the empty calculations were the Universal Force-Field (UFF) [41] (with fixed metal sites) and its extension specifically designed for Metal-Organic Frameworks UFF4MOF [42].

## 4. Conclusions

In post-synthetic modification of MOF linkers geometrical considerations are crucial for the final performance in a wide variety of applications such as energy transfer, optics, and mechanical properties. So far, however, no high-throughput screening method exists to optimize the geometrical design in the modification of MOF linkers. In this paper we present a screening method to post-synthetically crosslink MOF structures using any desired cross-linker molecule. The screening tool works independent of the chemistry involved in the post-synthetic functionalization and can be easily adjusted to other modification tasks such as MOF retrofitting or optimal functional group combinations in multivariant MOFs. The obtained results demonstrate great fitting accuracy and indicate possible combinations of MOF and cross-linker for any one of the investigated cross-linkers (CL1–CL6). The Hits for candidates CL3, CL4, and CL5 represent examples where the cross-linking would result in highly ordered MOF-COF hybrid structures. In addition, the screening tool also identified candidate structures for ship-in-a-bottle approaches (CL5) and mechanically interlocked cross-linkers, demonstrating the wide scope of the presented method. Still, it is critical to find a good balance between strict filtering parameters (to minimize false positive hits but eventually missing some hits) and more flexible thresholds (which identifies more possible hits while increase false positive hits). Currently, there is no definition of a standardized test set to quantify such parameter impact. Such a standardized test set would also allow to add more complexity to the search algorithms by filtering common pitfalls. Another great addition to the screening would be a quantitative screening of the candidates for physical properties, such as mechanical stability. In its current state, however, the results are a useful tool to first identify promising candidates before starting to synthesize the corresponding molecules and to synthesize the corresponding MOFs. Further analysis and simulation studies via MD or DFT of the screened candidates may also differentiate between good and bad cases and are definitely worth a thought.

## Abbreviations

The following abbreviations are used in this manuscript:

Metal-Organic Framework	MOF
Surface Mounted MOF	SURMOF
Covalent-Organic Framework	COF
Cross-Linker	CL
Molecular Dynamics	MD
High Performance Computing	HPC
Crystallographic Information Framework	CIF
Benzendicarboxylic Acid	bdc
Computation-Ready Experimental Metal-Organic Framework	CoRE MOF
